# Social, Emotional, and Existential Loneliness: A Test of the Multidimensional Concept

**DOI:** 10.1093/geront/gnaa082

**Published:** 2020-06-30

**Authors:** Theo G van Tilburg

**Affiliations:** Department of Sociology, Vrije Universiteit Amsterdam, The Netherlands

**Keywords:** Analysis, Factor analysis, Measurement, Social isolation

## Abstract

**Background and Objectives:**

Since the 1980s, most researchers have agreed on the concept of social and emotional loneliness as an unacceptable and negatively experienced discrepancy between realized and desired interpersonal relationships. For other researchers, existential loneliness stems from the realization that a human being is fundamentally alone, with the accompanying emptiness, sadness, and longing. This article examines whether instruments to measure these conceptualizations indicate a multidimensional concept.

**Research Design and Methods:**

The 2019 observation of the Longitudinal Aging Study Amsterdam (*N* = 1,316; aged 61–101 years; 52% women) included five direct questions about loneliness, the 11-item de Jong Gierveld social and emotional loneliness scale, and 14 items from the translated Existential Loneliness Questionnaire. Confirmatory factor analysis was conducted in Mplus.

**Results:**

Five factors were observed: direct questions, social and emotional loneliness, and loneliness in relationships and meaninglessness in life. The intercorrelations among all five factors were positive. Emotional loneliness correlated most strongly with direct questions.

**Discussion and Implications:**

Loneliness is multifaceted and means that one is not embedded in a personal network, misses closeness and intimacy, and lacks meaning in life. The emotional loneliness items most closely represent what people mean when they report loneliness.

Since the 1980s, most researchers have agreed on the concept of loneliness as a feeling of deprivation related to interpersonal relationships. Perlman and Peplau (1981, p. 31) defined loneliness as “the unpleasant experience that occurs when a person’s network of social relations is deficient in some important way, either quantitatively or qualitatively.” Other researchers have defined existential loneliness “as an intolerable emptiness, sadness, and longing, that results from the awareness of one’s fundamental separateness as a human being” (Ettema et al., 2010, p. 142). The two definitions and, as will be shown, their measuring instruments not only use similar terms, but also highlight a lack of social connection, albeit in a very different way. This article examines whether the instruments for measuring these conceptualizations indicate a multidimensional concept.

## The Concepts of Social, Emotional, and Existential Loneliness

Loneliness is a negative feeling related to loss and disappointment. It is the outcome of a process in which a person weighs up his or her existing personal relationships against his or her own wishes and social expectations with regard to relationships. If the social network is too small or the relationships are of insufficient quality, the person often feels lonely (de Jong Gierveld, 1978; Perlman & Peplau, 1981). This has been the most widely used conceptualization of loneliness in research. Two basic types of loneliness are social and emotional loneliness (Weiss, 1973). Social loneliness originates from the absence of a broader group of contacts or an engaging social network. Emotional loneliness originates from the absence of an intimate figure or a close emotional attachment. Social and emotional loneliness often occur due to reduced social activities (Aartsen & Jylhä, 2011) or loss of the partner (Utz et al., 2014), among other situations.

In a literature review, Bolmsjö et al. (2019) distinguished several key aspects of existential loneliness: not connecting with others and the world outside, alienation, feelings of isolation, emptiness, and abandonment. Additionally, mortality-related fears were identified to be associated with this type of loneliness, including the fear of disappearing from the earth, the fear of being forgotten, and the fear of dying. A further examination of this concept leads to two considerations.

First, the concept of existential loneliness needs to be sharpened and more clearly distinguished from social and emotional loneliness. For example, a negative evaluation of not connecting with others overlaps with social loneliness. Feelings of isolation, emptiness, and abandonment are aspects of emotional loneliness.

Second, social and emotional loneliness happen to somebody; people do not seek loneliness voluntarily. These types of loneliness are felt as social pain (Cacioppo et al., 2006). In contrast, some scholars see existential loneliness as something that can be appreciated positively; one finds strength in being solitary, although it takes effort to realize this situation. For Moustakas (1961), existential loneliness is inherent in human existence, such that everyone can identify as a solitary individual. People retreat from the social world to create a self-identity and to live a genuine life. Thus, in life’s most intimate and important moments, someone is an individual and is always alone. Loneliness can be a creative force among people who create music or inventions on their own. Ellison (1978) has described the separation by which humans give meaning to life and to their relationship with God. For Ettema et al. (2010), existential loneliness consists of negative feelings of nothingness that alternate and amalgamate with being in a process of inner growth. In this article, we focus on existential loneliness as a negative experience, and we aim to use the concept of solitude for when people want to be alone with positive purposes (Lay et al., 2020).

Existential loneliness is often studied in patients with severe illness (Tarbi & Meghani, 2019) and end-of-life situations (Boston et al., 2011). Casey and Holmes (1995) studied older people residing in nursing homes. After living a fulfilling and often busy life, these people are left without meaningful roles (Hupkens et al., 2018). Rosedale (2007) studied people with severe illnesses who realize their own mortality. Cherry and Smith (1993) examined loneliness in AIDS patients and observed that patients experienced inescapable separateness from others.

Existential loneliness differs from social and emotional loneliness in two ways. First, social and emotional loneliness are associated with a lack of meaningful social relationships and a lack of social companionship. Existential loneliness is the result of a broader separation related to the nature of existence and, in particular, a lack of meaning in life. An individual may be in the company of others but experience existential loneliness (Larsson et al., 2019). Second, social and emotional loneliness can be overcome by improving the quality of the network of relationships or by adjusting the level of aspiration (Rook & Peplau, 1982). Existential loneliness, on the other hand, has no permanent remedy according to the phenomenological approach (Mayers & Svartberg, 2001).

## The Measurement of Loneliness

Loneliness is often measured with a single, direct question. Such a question, for example, “Do you feel lonely?,” is simple to use, appears to be acceptable to respondents, reflects loneliness as understood by the respondent, and provides an easy way to assess the prevalence of loneliness (Jylhä & Saarenheimo, 2010; van Tilburg & de Jong Gierveld, 1999; Victor et al., 2005). However, the use of a direct question presupposes that the respondents have a common understanding of the term “loneliness” and that their understanding encompasses the whole theoretical concept. A single item does not provide information on the relevance of social, emotional, and existential aspects of loneliness. Because of the social stigma of loneliness (Lau & Gruen, 1992), people who are not seen as lonely by others may find it difficult to admit their loneliness as an answer to a direct question. Finally, the psychometric quality of a single question cannot be determined.

Alternatively, loneliness can be measured with a scale including statements that relate to loneliness but avoid the term “loneliness” or similar wording. The UCLA scale (Russell, 1996) and the de Jong Gierveld (DJG) scale (de Jong Gierveld & Kamphuis, 1985) are based on a conceptual framework of loneliness, in which different relational aspects and emotions relevant to the experience of loneliness are distinguished. Solano (1980) concluded that the UCLA scale appeared to identify a subjective lack of social companionship and is less sensitive to philosophically determined types of loneliness, such as existential loneliness. McWhirter (1990) and Hawkley et al. (2005) found various dimensions in the UCLA scale, but it is often considered a unidimensional measure. The DJG scale was developed as a unidimensional loneliness scale with both the social and emotional aspects of loneliness in mind. The homogeneity of the unidimensional scale proved to be modest at best. When searching for more homogeneous subscales, social and emotional loneliness factors emerged (de Jong Gierveld & van Tilburg, 1992; van Baarsen et al., 2001). The UCLA and DJG scales have rarely been studied in one sample. In a Dutch study, de Jong Gierveld and van Tilburg (1992) observed that the measurement of social loneliness was similar to that of the core dimension of the UCLA scale, which includes seven items. Another study (Penning et al., 2014) indicated that both scales were multidimensional, but the correlation between the scale scores was not reported.

While scales for social and emotional loneliness are widely used, there has been limited research into existential loneliness. Mayers et al. (2002) developed the Existential Loneliness Questionnaire (ELQ). This is the only scale we found that focuses on existential loneliness in survey research, with the exception of one studied among students in the 1970s (Belcher, 1973). The ELQ consists of 22 items and was found to be sufficiently internally consistent. Three items were specific to HIV, three were formulated conditionally, and two included a direct reference to loneliness. Gökdemir-Bulut and Bozo (2018) conducted two studies on the ELQ. They found three factors, that is, loneliness in social ties, loneliness in close relationships, and finding meaning in life; it appears that the first and second factors relate to social and emotional loneliness.

## The Current Study

As far as we know, no research has examined whether existential loneliness should be distinguished from social and emotional loneliness. The current research analyzes data from older respondents who answered items from the DJG and ELQ scales, along with direct loneliness questions. It tests whether social, emotional, and existential loneliness are related but distinctive dimensions of loneliness that are associated with direct measurements of loneliness. We also aim to determine whether existential loneliness is indeed a negative feeling.

To support the validity of the loneliness scales, we look for the association of various characteristics with the three types of loneliness. We expect to find that men and women have similar levels of loneliness scales scores (Maes et al., 2019) but that women will report more loneliness on the direct questions (Borys & Perlman, 1985). We also expect that age will correlate positively with all instruments (Luhmann & Hawkley, 2016), as will being without a partner, having a smaller network, not having daily network contact, and having poor health because unhealthy people have little social participation (de Jong Gierveld et al., 2018). Religiosity is linked to lower levels of loneliness (Lauder et al., 2006). Religious people are more involved than others in church-based social contacts and organizations in the community (Rote et al., 2013). In the current study, validity testing can go both ways. When there is a strong core in the concept of loneliness, we can establish that measurements of social, emotional, and existential loneliness are similarly associated with a number of antecedents. When multiple dimensions in loneliness need to be distinguished, we can establish that the measurements are differently associated with the antecedents.

## Design and Methods

### Respondents

Data were taken from the Longitudinal Aging Study Amsterdam (Huisman et al., 2011). This program employed samples of men and women born between 1908 and 1957 taken from the population registers of the cities of Amsterdam, Zwolle, and Oss, and six surrounding small municipalities in 1992, with additions in 2002 and 2012. The response rate at baseline was 63%. Follow-up interviews were conducted every 3 or 4 years. For each follow-up, an average of 82% of respondents were reinterviewed.

Data collection on 1,701 men and women was completed in 2019. We excluded 110 participants for whom data were collected from an interview with a proxy and 198 respondents interviewed by phone with a short questionnaire including only six social and emotional loneliness items. The DJG scale and three direct questions were part of the first, general face-to-face interview. One respondent was unable to participate in a full interview due to incapacity and did not answer the loneliness questions. The ELQ was part of a subsequent medical interview, which was conducted by another interviewer an average of 32 days after the initial interview. A total of 76 respondents from the first interview did not participate in the second interview. Thus, data from 1,316 respondents were analyzed. Their age varied between 61 and 101 years (*M* = 73.0), and 52% were women. Migrants were underrepresented. Most respondents were of Dutch origin; 3% were born in another Western country, and 2% were born in a non-Western country. Almost all respondents lived independently; 1% lived in a nursing home.

### Measures

Table 1 displays all loneliness questions and response options. Three direct loneliness questions were asked: a self-rating; an item assessing whether respondents “sometimes feel lonely” (de Jong Gierveld, 1984); and “During the past week I felt lonely,” which is adapted from the Center for Epidemiologic Studies Depression scale (Radloff, 1977). Two ELQ items that asked respondents whether they feel “lonely” and “alone” were also treated as direct questions.

**Table 1.** Loneliness Items, Mean and Loneliness Scores, Homogeneity, and Factor Loadings in the Five-Factor Model

						Factor loadings	
Nr.^a^	Item^b^		*M* ^c^	Lonely^d^	*H* ^e^	Estimate^f^	*R* ^2^
*Direct questions*					0.75		
—	If we were to classify people into not lonely, moderately lonely, strongly lonely, very strongly lonely, what would you attribute yourself to?^g^	Als we de mensen zouden indelen in niet eenzaam, matig eenzaam, sterk eenzaam, zeer sterk eenzaam, waar zou u zich dan nu toe rekenen?		0.21	0.73	1	0.91
—	I sometimes feel lonely^h^	Ik voel me soms wel eens eenzaam		0.25	0.78	0.96	0.84
—	During the past week I felt lonely^i^	De afgelopen week voelde ik me eenzaam		0.19	0.70	0.93	0.78
8	I feel lonely^j^	Ik voel mij eenzaam		0.09	0.81	1.00	0.91
21	I feel alone^j^	Ik voel me alleen		0.10	0.75	0.95	0.82
*Social loneliness* ^h^					0.51		
1	There is always someone I can talk to about my day-to-day problems†	Er is altijd wel iemand in mijn omgeving bij wie ik met mijn dagelijkse probleempjes terecht kan	1.17	0.13	0.46	1	0.68
4	There are plenty of people I can lean on when I have problems†	Er zijn genoeg mensen op wie ik in geval van narigheid kan terugvallen	1.14	0.12	0.52	0.96	0.63
7	There are many people I can trust completely†	Ik heb veel mensen op wie ik volledig kan vertrouwen	1.34	0.26	0.55	0.90	0.56
8	There are enough people I feel close to†	Er zijn voldoende mensen met wie ik me nauw verbonden voel	1.22	0.18	0.50	1.00	0.68
11	I can call on my friends whenever I need them†	Wanneer ik daar behoefte aan heb kan ik altijd bij mijn vrienden terecht	1.19	0.15	0.49	1.03	0.73
*Emotional loneliness* ^h^					0.53		
2	I miss having a really close friend	Ik mis een echt goede vriend of vriendin	1.27	0.18	0.52	1	0.63
3	I experience a general sense of emptiness	Ik ervaar een leegte om me heen	1.19	0.14	0.57	1.16	0.85
5	I miss the pleasure of the company of others	Ik mis gezelligheid om me heen	1.22	0.16	0.57	1.15	0.83
6	I find my circle of friends and acquaintances too limited	Ik vind mijn kring van kennissen te beperkt	1.27	0.19	0.49	0.99	0.61
9	I miss having people around me	Ik mis mensen om me heen	1.26	0.18	0.53	1.03	0.67
10	I often feel rejected	Vaak voel ik me in de steek gelaten	1.08	0.06	0.52	1.04	0.68
*Existential loneliness in relationships* ^j^					0.30		
6	I am surrounded by strangers I cannot connect with	Ik ben omringd door vreemden met wie ik geen contact kan leggen	1.78	0.11	0.34	1	0.37
12	I feel I have people I can trust and rely on if I need them†	Ik heb mensen waarop ik kan vertrouwen en rekenen wanneer ik daar behoefte aan heb	1.64	0.06	0.33	1.06	0.42
16	I stay in bad relationships too long in order not to be alone	Ik blijf te lang in een slechte relatie omdat ik niet alleen wil zijn	1.59	0.06	0.30	0.97	0.35
23	I mean something to others†	Ik beteken iets voor andere mensen	2.13	0.23	0.23	0.80	0.24
24	Important relationships have ended or become weaker	Belangrijke contacten zijn weggevallen of verwaterd	2.55	0.45	0.31	1.01	0.38
30	No one else in the world can understand my feelings	Niemand kan mijn gevoelens begrijpen	2.14	0.27	0.32	0.97	0.35
31	My world seems so different from everybody else’s	Mijn wereld is totaal anders dan die van andere mensen	2.16	0.25	0.26	0.81	0.24
*Existential loneliness: meaninglessness in life* ^j^					0.34		
1	I am happy with the way I have lived my life†	Ik ben gelukkig over hoe ik mijn leven heb geleid	1.91	0.16	0.35	1	0.41
3	There is a purpose to my life†	Mijn leven heeft een bepaalde bedoeling	2.47	0.43	0.22	0.53	0.12
18	I feel helpless	Ik voel me hulpeloos	1.52	0.05	0.42	1.27	0.66
25	I feel at the mercy of the world	Ik voel mij machteloos tegenover de wereld	2.25	0.33	0.30	0.87	0.31
26	I feel dead	Het voelt alsof ik dood ben	1.38	0.02	0.42	1.27	0.66
27	The universe is full of meaning†	Deze wereld biedt vele mogelijkheden	2.12	0.23	0.31	0.77	0.24
29	I feel that there is little point to life	Het leven heeft weinig zin	1.67	0.10	0.44	1.20	0.60

*Note:* DJG = de Jong Gierveld; ELQ = Existential Loneliness Questionnaire.

^a^Item numbers are taken from the DJG and ELQ scales.

^b^Scores are reversed for positively phrased items marked with †.

^c^Range for social and emotional loneliness items is 1–3; for existential loneliness 1–5.

^d^Lonely: the proportion that answered affirmatively in the loneliness direction or answered in the middle category.

^e^The scalability coefficient *H* (homogeneity) is presented for the scale as a whole and for individual items.

^f^All estimates *p* < .001.

^g^Asked in the first interview. Response options: “not lonely,” “moderately lonely,” “strongly lonely,” and “very strongly lonely.”

^h^Asked in the first interview. Response options: “no,” “more or less,” and “yes.”

^i^Asked in the first interview. Response options: “rarely or never,” “some of the time,” “occasionally,” and “mostly or always.”

^j^Asked in the second interview. Response options: “no!,” “no,” “more or less,” “yes,” and “yes!”

From the DJG scale, five positively phrased items and six negatively phrased items measure the intensity of deprivation, which is considered to be the essence of loneliness. The subscales reflect social loneliness and emotional loneliness, respectively.

Existential loneliness was measured by 14 ELQ items. We excluded three HIV items and three conditionally formulated items. We performed a translation from English into Dutch, followed by back translation, using two translators in both steps (Beaton et al., 2000).

Marital and partner status was assessed by three questions. Network members with whom there was regular contact and who were important to the respondent were identified by name across seven domains (van Tilburg, 1998). We derived two characteristics: personal network size (not counting the partner) and being in daily contact with someone in the network. Self-rated health was measured using the question “How is your health in general?” (poor to very good). The frequency of church attendance was assessed on a six-point scale from “never” to “once a week or more.”

### Procedure

The scores on the five direct questions were recoded so that the answers “not lonely,” “rarely or never,” “no,” and “no!” indicate the absence of loneliness and the other answers indicate loneliness; it was assumed that the middle category “more or less” indicates loneliness (de Jong Gierveld & Kamphuis, 1985). We investigated the psychometric properties of the scales. Homogeneity (*H*), computed by Mokken scale analysis, was indicated by the correlation between the item scores (scale lower limit is 0.30, indicating weak homogeneity; 0.40–0.50 indicates medium homogeneity; 0.50 or higher shows strong homogeneity; Sijtsma & van der Ark, 2017). Reliability describes the interrelationship in terms of the number of items (lower limit 0.80; Nunnally & Bernstein, 1994).

We applied confirmatory factor analysis in Mplus (Muthén & Muthen, 2017). Item scores were treated as having categorical (direct questions) and ordinal (all other) measurement levels; latent variables were continuous. First, we tested whether there are five factors underlying the 30 items. The five factors are direct measurements, social loneliness, emotional loneliness, and the existential aspects “loneliness in relationships” and “meaninglessness in life,” which are derived from the work of Gökdemir-Bulut and Bozo (2018). Next, to investigate the commonality of the five factors, we tested a unidimensional, second-order, and bifactor model (Canivez, 2016). To test the model fit, we used χ ^2^/*df*, the comparative fit index (CFI), the Tucker–Lewis index (TLI), and the root mean square error of approximation (RMSEA): χ ^2^/*df* <3, CFI and TLI ≥0.95, and RMSEA ≤0.07 indicate an acceptable model fit (Hooper et al., 2008). The model was estimated using the full information maximum likelihood method. We present unstandardized factor loadings herein. Correlations between factors were corrected for attenuation. Estimated factor scores were derived to create individual loneliness scale scores. We also computed Pearson correlations between loneliness scale scores and the antecedents of loneliness. Differences in strength between loneliness scores were tested with the *z*-statistic (Lee & Preacher, 2013).

## Results

There were few missing data. One respondent did not answer five items, and 16 respondents did not answer ELQ item 16. The frequency of loneliness as indicated by the 30 items varied considerably (Table 1). Of the three direct questions in the first interview, between 19% and 25% of the respondents reported being lonely. Only approximately 10% reported loneliness using the two direct ELQ questions. For the social loneliness items, an average of 17% of respondents answered affirmatively to indicate loneliness (including answers in the middle category). Many respondents answered negatively to the item “There are many people I can trust completely” (DJG 7). On average 15% reported emotional loneliness. Few respondents answered affirmatively to the item “I often feel rejected.” On average 20% reported existential loneliness in relationships. Compared to other items, respondents answered more affirmatively on ELQ item 12, that is, “can trust and rely,” indicating low levels of loneliness; however, a differently worded item on trust from the DJG scale (item 7) indicated higher levels of loneliness. There were a high number of affirmative responses to the item “Important relationships have ended or become weaker.” Finally, the average for meaninglessness in life was 19%. There were few affirmative answers to the item “I feel dead.” Many respondents disagreed with the statement “There is a purpose in my life.”

The homogeneity of loneliness as measured by direct questions was strong (0.75; Table 1), and the reliability was sufficient (Kuder-Richardson 20 [KR-20] = 0.86). For social and emotional loneliness, the homogeneity was strong (0.51 and 0.53, respectively), and the reliability was (almost) sufficient (KR-20 = 0.79 and 0.84, respectively). The two existential loneliness scales are weakly homogeneous (0.30 and 0.34) and have low reliability (Cronbach’s alpha = 0.69 and 0.73).

The five-factor model had the best fit and three of the four indices indicated that the model fit the data (Table 2). For each factor, one item served as a reference with a factor loading of 1; for the direct questions factor, the reference item was the self-rated item (Table 1). The lower estimated factor loadings for three items (“sometimes lonely,” “last week lonely,” and “feel alone”) indicate a weaker correlation between the item and the latent factor compared to the other two items. The *R*^2^ represents these correlations. The high item–factor correlations for direct questions and social and emotional loneliness factors and the lower correlations for the two existential loneliness factors are congruent with the differences in homogeneity and reliability reported previously.

**Table 2.** Fit of Four Models in Confirmatory Factor Analysis

Model	χ ^2^	*df*	χ ^2^/*df*	CFI	TLI	RMSEA
Five-factor	1,452.3	395	3.7	0.961	0.957	0.045
Unidimensional	4,237.7	405	10.5	0.860	0.849	0.085
Second-order	2,734.1	400	6.8	0.915	0.907	0.067
Bifactor	2,750.6	375	7.3	0.913	0.899	0.069

*Note:* CFI = comparative fit index; RMSEA = root mean square error of approximation; TLI = Tucker–Lewis index.

Attenuation-corrected correlations between the five loneliness scale scores varied between 0.32 and 0.82 (Table 3). The highest correlation was found between the direct questions and emotional loneliness. Correlations among the two existential loneliness scales and the other factors were relatively low.

**Table 3.** Observed Correlations and Correlations Corrected for Attenuation^a^ Between Loneliness Scale Scores^b^

	Direct questions	Social loneliness	Emotional loneliness	Existential loneliness in relationships
Social loneliness	0.48			
	*0.58*			
Emotional loneliness	0.70	0.47		
	*0.82*	0.58		
Existential loneliness in relationships	0.36	0.36	0.32	
	*0.46*	*0.48*	*0.42*	
Existential loneliness: meaninglessness in life	0.38	0.29	0.30	0.37
	*0.48*	*0.38*	*0.32*	*0.51*

^a^Correlations corrected for attenuation in italic.

^b^All observed correlations *p* < .001.

Most respondents (63%) lived with their spouse; fewer than 1% lived independently while their spouse lived in a nursing home; 3% were not married and lived with a partner; 5% had a partner who lived outside of the household; and 29% did not have a partner. Most respondents had extensive personal networks: fewer than 1% had no or only one tie, and 91% had six or more ties; the average network size was 16.5 ties. In addition to daily contact with the partner with whom one lived (66%), 18% had daily contact with someone else, and 15% had no daily network contact. Health was rated as good or very good by 68%. The frequency of church attendance was monthly or more frequently for 23% of the respondents. Correlations between the loneliness scales and gender (with the exception of emotional loneliness), age, network characteristics, and health were in the direction similar to the directions reported in the literature (Table 4). The correlations were generally modest and there were few differences between the five scales. Most striking was that the correlation with living with spouse or partner was relatively high for the direct questions and emotional loneliness. Loneliness did not correlate with the frequency of church attendance.

**Table 4.** Correlations Between Loneliness Scale Scores and Antecedents of Loneliness

	Direct questions			Social loneliness			Emotional loneliness			Existential loneliness in relationships			Existential loneliness: meaninglessness		
Female (vs. male)	0.11	***	^a^	−0.01		^a^	0.08	**		0.01			0.04		
Age (61–101 years)	0.26	***		0.25	***		0.26	***		0.32	***		0.31	***	
Living with spouse or partner (vs. not)	−0.44	***	^abc^	−0.29	***	^a^	−0.42	***	^de^	−0.28	***	^bd^	−0.27	***	^ce^
Network size (0–78)	−0.25	***	^a^	−0.35	***	^a^	−0.28	***		−0.32	***		−0.28	***	
Daily network contact (vs. not)	−0.31	***		−0.27	***		−0.31	***		−0.24	***		−0.22	***	
Self-rated health (1–5)	−0.24	***		−0.20	***		−0.22	***		−0.27	***		−0.29	***	
Church attendance frequency (1–6)	−0.02			−0.05			−0.03			−0.03			−0.02		

*Note:* Correlations marked with the same letter in superscript differ (*p* < .001).

**p < .01; ***p < .001.

## Discussion and Implications

By applying the translated ELQ among older Dutch adults, we found evidence for multiple dimensions of loneliness in addition to social and emotional loneliness. Gökdemir-Bulut and Bozo (2018) tested the ELQ and assumed three factors. Because we excluded a number of items for different reasons, we combined two factors into one dimension, namely, “existential loneliness in relationships.” This ELQ dimension focuses on social bonding in relationships and has similarities with social loneliness (e.g., item 12 with DJG item 7). However, this ELQ dimension was only modestly related to social loneliness. This modest association may be due to addressing different aspects of loneliness in the other items and due to different wordings and mode effects, such as the use of two different interviews and different numbers of response options. The congruence of “existential loneliness in relationships” and social and emotional loneliness needs to be further investigated. We conclude that the ELQ subscale on relationships does not contribute sufficiently to the conceptualization of loneliness. In addition, the subscale is weakly homogeneous and insufficiently reliable. The third factor distinguished by Gökdemir-Bulut and Bozo relates to a differential conceptualization that we have labeled “meaninglessness in life.” The positive correlations among existential loneliness scale scores, the direct question scores, and social and emotional loneliness show that existential loneliness as measured with the ELQ is not a positive and rewarding experience. However, we did not address the fact that individuals sometimes seek solitude (Lay et al., 2020) or think they are better off alone (Birditt et al., 2019), and we did not measure the “desire for aloneness” (Leung, 2015).

The lack of fit of the unidimensional, second-order, and bifactor models and the modest correlations between the five factors, especially between social and emotional loneliness on the one hand and existential loneliness on the other, suggest that the various instruments address many different aspects. We can see the collection of these aspects as representative for one broad multidimensional theoretical concept, or, alternatively, these aspects do not have enough in common to be seen as dimensions of one sharply defined concept of loneliness and represent two concepts, namely, “loneliness” and “meaning in life” (Brandstätter et al., 2012), each with their own definition. The three dimensions can also be interpreted to cover three aspects of the life experience (Steptoe et al., 2015): how satisfied people are with their lives (evaluative), feelings and moods (hedonistic), and assessing giving meaning to life (eudemonic). However, unlike Steptoe et al.’s findings, we did not find clearly distinct antecedents associated with the loneliness dimensions. This may indicate a commonality: social, emotional, and existential aspects are all negative loneliness feelings pointing to a lack of connectedness with people and life, which are associated in the same way to a number of loneliness antecedents. On the basis of the antecedents, the three dimensions cannot therefore be sufficiently distinguished from each other to be treated as a standalone phenomenon. In future research, we propose using 18 questions that measure three types of loneliness. The existential scale could be improved.

We also included direct questions on loneliness in our analyses. The strong homogeneity of the factor with direct questions may be related to the repetition of the word “loneliness” (or variants) in four of the five items. Balancing the advantages and disadvantages of direct questions compared to scales, many researchers prefer the use of scales. The use of a direct question can be of additional value when a strong correlation is observed with a loneliness scale score to confirm the validity of the scale (Victor et al., 2005). The high correlation we observed between the scores for the direct questions and the emotional loneliness scale scores suggests that the evaluations and feelings included in the six emotional loneliness items most closely represent what people mean when they report loneliness. A researcher who prefers to use only one direct question does not know which type of loneliness the question measures, or in the best case, it most likely measures emotional loneliness in an unreliable way.

It is now widely recognized that loneliness is related to public health, for example, due to increased premature mortality among lonely people (Holt-Lunstad et al., 2015). Therefore, policy makers are interested in the prevalence of loneliness, and practitioners treating clients who may be lonely need to assess whether loneliness is the problem. Scale ratings of loneliness indicate the severity of loneliness but do not indicate the prevalence of loneliness or whether a person is lonely. Providing a cutoff score of a loneliness scale may solve this problem but also needs a reference that can be found in answers on direct loneliness questions (van Tilburg & de Jong Gierveld, 1999). We found that the frequency of signs of loneliness varies greatly between items. This may have to do with the period covered in the items (e.g., last week or undefined), the wording of the items (e.g., trusting many people completely or having a trustee), the different number of response options (three to five), and the context of the interview (i.e., general or medical). This suggests that both the basis for determining loneliness prevalence and the likelihood of an individual being assessed as lonely vary based on the design of the research. However, we observed strong homogeneity among the answers to the five direct questions. This offers opportunities for an improved way to estimate the prevalence of loneliness and to assess individuals. Answers to various questions can be averaged, and this average can be used to determine appropriate cutoff scores.

We want to draw attention to some design issues in addition to those addressed previously. First, respondents participated in two long interviews. The sample does not include many older people who are in a serious situation, such as severe illness or the end of life, which is the focus of a number of studies on existential loneliness. Second, there was a time between the two interviews, which contributed to participants avoiding response sets and other ways in which offering many questions on one topic can affect the outcome of the research. At the same time, the respondent’s situation may have changed between the two interviews, which may have contributed to the relatively low correlation between the DJG scores (assessed in the first interview) and the ELQ scores (assessed in the second interview). However, we also note that the factor of direct questions, of which three were asked in the first interview and two were asked in the second interview, had very good psychometric properties. This suggests that the interval between the two interviews did not strongly influence our results. Finally, we did not look in detail at the quality of the scales and did not investigate whether removing items leads to more homogeneous scales.

To conclude, we explored the dimensions of loneliness and found that social, emotional, and existential aspects were relevant. These were equally related to the antecedents of loneliness. Direct questions may be of additional value in assessing loneliness among older adults.

## Funding

The Longitudinal Aging Study Amsterdam is supported by a grant from the Netherlands Ministry of Health, Welfare and Sport, Directorate of Long-Term Care.

## Conflict of Interest

None declared.

## References

[CIT0001] Aartsen, M. J., & Jylhä, M. (2011). Onset of loneliness in older adults: Results of a 28-year prospective study. European Journal of Ageing, 8(1), 31–38. doi:10.1007/s10433-011-0175-7 21475393 PMC3047676

[CIT0002] Beaton, D. E., Bombardier, C., Guillemin, F., & Ferraz, M. B. (2000). Guidelines for the process of cross-cultural adaptation of self-report measures. Spine, 25(24), 3186–3191. doi:10.1097/00007632-200012150-00014 11124735

[CIT0003] Belcher, M. J. (1973). The measurement of loneliness: A validation study of the Belcher Extended Loneliness Scale (BELS) [PhD dissertation]. Illinois Institute of Technology, Chicago.

[CIT0004] Birditt, K. S., Manalel, J. A., Sommers, H., Luong, G., & Fingerman, K. L. (2019). Better off alone: Daily solitude is associated with lower negative affect in more conflictual social networks. The Gerontologist, 59(6), 1152–1161. doi:10.1093/geront/gny060 29924314 PMC6858825

[CIT0005] Bolmsjö, I., Tengland, P. A., & Rämgård, M. (2019). Existential loneliness: An attempt at an analysis of the concept and the phenomenon. Nursing Ethics, 26(5), 1310–1325. doi:10.1177/0969733017748480 29471724

[CIT0006] Borys, S., & Perlman, D. (1985). Gender differences in loneliness. Personality & Social Psychology Bulletin, 11, 63–74. doi:10.1177/0146167285111006

[CIT0007] Boston, P., Bruce, A., & Schreiber, R. (2011). Existential suffering in the palliative care setting: An integrated literature review. Journal of Pain and Symptom Management, 41(3), 604–618. doi:10.1016/j.jpainsymman.2010.05.010 21145202

[CIT0008] Brandstätter, M., Baumann, U., Borasio, G. D., & Fegg, M. J. (2012). Systematic review of meaning in life assessment instruments. Psycho-Oncology, 21(10), 1034–1052. doi:10.1002/pon.2113 22232017

[CIT0009] Cacioppo, J. T., Hawkley, L. C., Ernst, J. M., Burleson, M., Berntson, G. G., Nouriani, B., & Spiegel, D. (2006). Loneliness within a nomological net: An evolutionary perspective. Journal of Research in Personality, 40(6), 1054–1085. doi:10.1016/j.jrp.2005.11.007

[CIT0010] Canivez, G. L. (2016). Bifactor modeling in construct validation of multifactored tests: Implications for understanding multidimensional constructs and test interpretation. In K. Schweizer & C. DiStefano (Eds.), Principles and methods of test construction: Standards and recent advancements (pp. 247–271). Hogrefe.

[CIT0011] Casey, M. S., & Holmes, C. A. (1995). The inner ache: An experiential perspective on loneliness. Nursing Inquiry, 2(3), 172–179. doi:10.1111/j.1440-1800.1995.tb00168.x 7664162

[CIT0012] Cherry, K., & Smith, D. H. (1993). Sometimes I cry: The experience of loneliness for men with AIDS. Health Communication, 5(3), 181–208. doi:10.1207/s15327027hc0503_3

[CIT0013] de Jong Gierveld, J. (1978). The construct of loneliness: Components and measurement. Essence, 2(4), 221–237.

[CIT0014] de Jong Gierveld, J. (1984). Eenzaamheid: Een meersporig onderzoek [Loneliness: A multimethod approach]. Van Loghum Slaterus.

[CIT0015] de Jong Gierveld, J., & Kamphuis, F. H. (1985). The development of a Rasch-type loneliness-scale. Applied Psychological Measurement, 9, 289–299. doi:10.1177/014662168500900307

[CIT0016] de Jong Gierveld, J., & van Tilburg, T. G. (1992). Triangulatie in operationaliseringsmethoden [Triangulation in operationalisation methods]. In G. J. N. Bruinsma & M. A. Zwanenburg (Eds.), Methodologie voor bestuurskundigen: Stromingen en methoden (pp. 273–298). Coutinho.

[CIT0017] de Jong Gierveld, J., van Tilburg, T. G., & Dykstra, P. A. (2018). New ways of theorizing and conducting research in the field of loneliness and social isolation. In A. L. Vangelisti & D. Perlman (Eds.), The Cambridge handbook of personal relationships (pp. 391–404). Cambridge University Press.

[CIT0018] Ellison, C. W. (1978). Loneliness: A social–developmental analysis. Journal of Psychology and Theology, 6(1), 3–17. doi:10.1177/009164717800600101

[CIT0019] Ettema, E. J., Derksen, L. D., & van Leeuwen, E. (2010). Existential loneliness and end-of-life care: A systematic review. Theoretical Medicine and Bioethics, 31(2), 141–169. doi:10.1007/s11017-010-9141-1 20440564 PMC2866502

[CIT0020] Gökdemir-Bulut, B. P., & Bozo, Ö. (2018). The psychometric validity and reliability of the Turkish version of the Existential Loneliness Questionnaire. Current Psychology, 37(1), 401–413. doi:10.1007/s12144-016-9534-z

[CIT0021] Hawkley, L. C., Browne, M. W., & Cacioppo, J. T. (2005). How can I connect with thee? Let me count the ways. Psychological Science, 16(10), 798–804. doi:10.1111/j.1467-9280.2005.01617.x 16181443

[CIT0022] Holt-Lunstad, J., Smith, T. B., Baker, M., Harris, T., & Stephenson, D. (2015). Loneliness and social isolation as risk factors for mortality: A meta-analytic review. Perspectives on Psychological Science, 10(2), 227–237. doi:10.1177/1745691614568352 25910392

[CIT0023] Hooper, D., Coughlan, J., & Mullen, M. R. (2008). Structural equation modelling: Guidelines for determining model fit. Electronic Journal of Business Research Methods, 6(1), 53–60. doi:10.21427/D7CF7R

[CIT0024] Huisman, M., Poppelaars, J., van der Horst, M., Beekman, A. T., Brug, J., van Tilburg, T. G., & Deeg, D. J. (2011). Cohort profile: The Longitudinal Aging Study Amsterdam. International Journal of Epidemiology, 40(4), 868–876. doi:10.1093/ije/dyq219 21216744

[CIT0025] Hupkens, S., Machielse, A., Goumans, M., & Derkx, P. (2018). Meaning in life of older persons: An integrative literature review. Nursing Ethics, 25(8), 973–991. doi:10.1177/0969733016680122 30871429

[CIT0026] Jylhä, M., & Saarenheimo, M. (2010). Loneliness and ageing: Comparative perspectives. In D. Dannefer & C. Phillipson (Eds.), The Sage handbook of social gerontology (pp. 317–328). Sage.

[CIT0027] Larsson, H., Edberg, A. K., Bolmsjö, I., & Rämgård, M. (2019). Contrasts in older persons’ experiences and significant others’ perceptions of existential loneliness. Nursing Ethics, 26(6), 1623–1637. doi:10.1177/0969733018774828 29772961

[CIT0028] Lau, S., & Gruen, G. E. (1992). The social stigma of loneliness: Effect of target person’s and perceiver’s sex. Personality and Social Psychology Bulletin, 18(2), 182–189. doi:10.1177/0146167292182009

[CIT0029] Lauder, W., Mummery, K., & Sharkey, S. (2006). Social capital, age and religiosity in people who are lonely. Journal of Clinical Nursing, 15(3), 334–340. doi:10.1111/j.1365-2702.2006.01192.x 16466483

[CIT0030] Lay, J. C., Pauly, T., Graf, P., Mahmood, A., & Hoppmann, C. A. (2020). Choosing solitude: Age differences in situational and affective correlates of solitude-seeking in midlife and older adulthood. The Journals of Gerontology, Series B: Psychological Sciences and Social Sciences, 75(3), 483–493. doi:10.1093/geronb/gby044 29669095

[CIT0031] Lee, I. A., & Preacher, K. J. (2013). Calculation for the test of the difference between two dependent correlations with one variable in common [Computer software]. Retrieved from http://quantpsy.org

[CIT0032] Leung, L. (2015). Using tablet in solitude for stress reduction: An examination of desire for aloneness, leisure boredom, tablet activities, and location of use. Computers in Human Behavior, 48, 382–391. doi:10.1016/j.chb.2015.01.068

[CIT0033] Luhmann, M., & Hawkley, L. C. (2016). Age differences in loneliness from late adolescence to oldest old age. Developmental Psychology, 52(6), 943–959. doi:10.1037/dev0000117 27148782 PMC8015413

[CIT0034] Maes, M., Qualter, P., Vanhalst, J., van den Noortgate, W., & Goossens, L. (2019). Gender differences in loneliness across the lifespan: A meta-analysis. European Journal of Personality, 33(6), 642–654. doi:10.1002/per.2220

[CIT0035] Mayers, A. M., Khoo, S. T., & Svartberg, M. (2002). The existential loneliness questionnaire: Background, development, and preliminary findings. Journal of Clinical Psychology, 58(9), 1183–1193. doi:10.1002/jclp.10038 12209873

[CIT0036] Mayers, A. M., & Svartberg, M. (2001). Existential loneliness: A review of the concept, its psychosocial precipitants and psychotherapeutic implications for HIV‐infected women. British Journal of Medical Psychology, 74(4), 539–553. doi:10.1348/000711201161082 11780800

[CIT0037] McWhirter, B. T. (1990). Factor analysis of the revised UCLA loneliness scale. Current Psychology, 9(1), 56–68. doi:10.1007/bf02686768

[CIT0038] Moustakas, C. E. (1961). Loneliness. Prentice-Hall.

[CIT0039] Muthén, L. K., & Muthen, B. (2017). Mplus user’s guide: Statistical analysis with latent variables. Muthén & Muthén.

[CIT0040] Nunnally, J. C., & Bernstein, I. H. (1994). Psychometric theory. McGraw-Hill.

[CIT0041] Penning, M. J., Liu, G., & Chou, P. H. B. (2014). Measuring loneliness among middle-aged and older adults: The UCLA and de Jong Gierveld loneliness scales. Social Indicators Research, 118(3), 1147–1166. doi:10.1007/s11205-013-0461-1

[CIT0042] Perlman, D., & Peplau, L. A. (1981). Toward a social psychology of loneliness. In R. Gilmour & S. W. Duck (Eds.), Personal relationships in disorder (pp. 31–56). Academic Press.

[CIT0043] Radloff, L. S. (1977). The CES-D scale: A self-report depression scale for research in the general population. Applied Psychological Measurement, 1(3), 385–401. doi:10.1177/014662167700100306

[CIT0044] Rook, K. S., & Peplau, L. A. (1982). Perspectives on helping the lonely. In L. A. Peplau & D. Perlman (Eds.), Loneliness: A sourcebook of current theory, research and therapy (pp. 351–378). Wiley.

[CIT0045] Rosedale, M. (2007). Loneliness: An exploration of meaning. Journal of the American Psychiatric Nurses Association, 13(4), 201–209. doi:10.1177/1078390307306617

[CIT0046] Rote, S., Hill, T. D., & Ellison, C. G. (2013). Religious attendance and loneliness in later life. The Gerontologist, 53(1), 39–50. doi:10.1093/geront/gns063 22555887 PMC3551208

[CIT0047] Russell, D. W. (1996). UCLA Loneliness Scale (Version 3): Reliability, validity, and factor structure. Journal of Personality Assessment, 66(1), 20–40. doi:10.1207/s15327752jpa6601_2 8576833

[CIT0048] Sijtsma, K., & van der Ark, L. A. (2017). A tutorial on how to do a Mokken scale analysis on your test and questionnaire data. The British Journal of Mathematical and Statistical Psychology, 70(1), 137–158. doi:10.1111/bmsp.12078 27958642

[CIT0049] Solano, C. H. (1980). Two measures of loneliness: A comparison. Psychological Reports, 46(1), 23–28. doi:10.2466/pr0.1980.46.1.23 7367538

[CIT0050] Steptoe, A., Deaton, A., & Stone, A. A. (2015). Subjective wellbeing, health, and ageing. Lancet (London, England), 385(9968), 640–648. doi:10.1016/S0140-6736(13)61489-0 PMC4339610 25468152

[CIT0051] Tarbi, E. C., & Meghani, S. H. (2019). A concept analysis of the existential experience of adults with advanced cancer. Nursing Outlook, 67(5), 540–557. doi:10.1016/j.outlook.2019.03.006 31040052 PMC6764914

[CIT0052] Utz, R. L., Swenson, K. L., Caserta, M., Lund, D., & deVries, B. (2014). Feeling lonely versus being alone: Loneliness and social support among recently bereaved persons. The Journals of Gerontology, Series B: Psychological Sciences and Social Sciences, 69(1), 85–94. doi:10.1093/geronb/gbt075 PMC3894128 24056690

[CIT0500] van Baarsen, B., Snijders, T. A. B., Smit, J. H., & van Duijn, M. A. J. (2001). Lonely but not alone: Emotional isolation and social isolation as two distinct dimensions of loneliness in older people. Educational and Psychological Measurement, 61, 119–135. doi:10.1177/00131640121971103

[CIT0053] van Tilburg, T. G. (1998). Losing and gaining in old age: Changes in personal network size and social support in a four-year longitudinal study. Journal of Gerontology, 53B, S313–S323. doi:10.1093/geronb/53B.6.S313 9826973

[CIT0054] van Tilburg, T. G., & de Jong Gierveld, J. (1999). Reference standards for the loneliness scale. Tijdschrift voor Gerontologie en Geriatrie, 30(4), 158–163. 10486620

[CIT0055] Victor, C. R., Grenade, L., & Boldy, D. (2005). Measuring loneliness in later life: A comparison of differing measures. Reviews in Clinical Gerontology, 15(1), 63–70. doi:10.1017/S0959259805001723

[CIT0056] Weiss, R. S. (1973). Loneliness: The experience of emotional and social isolation. MIT Press.

